# Ketamine and Its Emergence in the Field of Neurology

**DOI:** 10.7759/cureus.27389

**Published:** 2022-07-28

**Authors:** Luis Rueda Carrillo, Klepper Alfredo Garcia, Nilufer Yalcin, Manan Shah

**Affiliations:** 1 Neurology, Augusta University Medical College of Georgia, Augusta, USA

**Keywords:** migraine, traumatic brain injury, status epilepticus, stroke, ketamine, psychoactive drug

## Abstract

The quest for a safe and effective anesthetic medication in the mid-20th century led to the discovery of CI-581, which was later named ketamine. Ketamine was labeled a “dissociative anesthetic” due to the state of sensory deprivation that it induces in the subjects receiving it. Although it enjoyed widespread use at the beginning of the Vietnam war, its use rapidly waned due to its psychedelic effect and it became more popular as a recreational drug, and in the field of veterinary medicine. However, as we gained more knowledge about its multiple sites of action, it has reemerged as a useful anesthetic/analgesic agent. In the last decade, the field of neurology has witnessed the growing use of ketamine for the treatment of several neurological conditions including migraine, status epilepticus, stroke, and traumatic brain injury (TBI). Ketamine acts primarily as a non-competitive N-methyl-D-aspartate (NMDA) receptor antagonist. The binding of ketamine to NMDA receptors leads to decreased frequency and duration of Ca^+2^ channel opening and thus inhibits glutaminergic transmission. This mechanism has proven to be neuroprotective in several neurological conditions. Ketamine does not increase intracranial pressure (ICP), and it maintains cerebral perfusion pressure (CPP) by increasing cerebral blood flow. Ketamine has also been shown to inhibit massive slow waves of neurological depolarizations called cortical spreading depolarizations (CSD), usually seen during acute neurological injury and are responsible for further neurological deterioration. Unlike other anesthetic agents, ketamine does not cause cardiac or respiratory suppression. All these favorable mechanisms and cerebral/hemodynamic actions have led to increased interest among clinicians and researchers regarding the novel uses of ketamine. This review will focus on the use of ketamine for various neurological indications.

## Introduction and background

The quest for a safer and more effective anesthetic in the mid-20th century led to the discovery of phencyclidine (PCP), which relied on the Nobel Prize-winning Grignard reaction. Although it was deemed a safe anesthetic, it often produced a prolonged condition of delirium and sensory deprivation during the postoperative recovery period [1,2]. The search for its short-active derivative led to the synthesis of the compound CI-581 in 1962, which was later renamed ketamine. Dr. Edward Domino and Dr. Guenter Corssen performed the first clinical study on ketamine, involving 20 male prisoners, describing its properties and effects. During their experiment, they identified that the volunteers remained in a state where they might appear awake, with preserved reflexes and the ability to protect their airways, although unable to respond to a sensory stimulus [3]. The term "dissociative anesthesia" was proposed to describe this mental state. Following further human trials in 1966, ketamine received approval from the Food and Drug Administration (FDA) in 1970 and was introduced to the general population with the trade name Ketalar [2].

During the 1970s, ketamine was used extensively for surgical anesthesia in the field during the Vietnam War. However, concerns were raised about the sense of delirium caused by the medication, leading to a decline in its use as a human anesthetic. In subanesthetic doses, ketamine was found to have a "psychedelic effect" [4]. The first reports of recreational misuse started to emerge as early as 1971 [5], and as the rave culture spread throughout the US and internationally, ketamine became a "club drug." As its popularity among young partygoers surged during the 1980s, ketamine became commonly known by various street names, including Vitamin K, Kit Kat, K-land, K-hole, CatValium, and special K. Eventually, ketamine fell out of favor in the medical community and became a schedule III drug.

Ketamine had a resurgence in its medical use around the 1990s as we gained more insight into its mechanism of action and efficacy [6,7]. Studies in the field of anesthesia have led to ketamine being recognized as a safe and effective option to treat postoperative pain, complex pain syndrome, and other neuropathic conditions. Furthermore, due to its neuromodulation effects, there has been a growing interest in its use in the treatment of many psychiatric disorders, including depression and post-traumatic stress disorder; researchers have also used ketamine as a model to study schizophrenia [2,7].

Over the past two decades, evidence has emerged about the efficacy of ketamine to treat multiple neurological conditions, including subarachnoid hemorrhages (SAH), traumatic brain injury (TBI), migraines, and refractory status epilepticus (RSE). This review will focus on its pharmacokinetics and pharmacodynamics, the effects of ketamine on the brain, and its current uses in neurology.

## Review

Pharmacology

**Figure 1:**
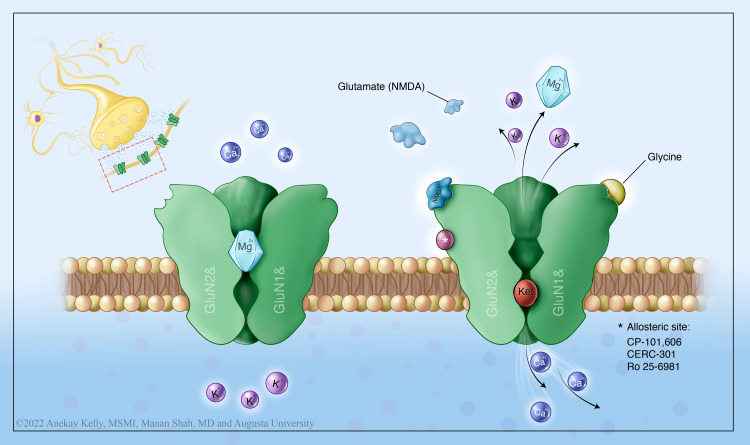
Ketamine's mechanism of action Illustration by Anekay Kelly, MSMI

Ketamine is a derivate of phencyclidine, with the formula 2-(o-chlorophenyl)-2-methyl-amino cyclohexane HCL (Figure 1). The asymmetric C2 carbon serves as a chiral center leading to two optical enantiomers: S(+) and R(-). (S)-ketamine enantiomer is a more potent anesthetic owing to its threefold higher affinity for N-methyl-D-aspartate (NMDA) receptor compared to (R)-ketamine [8], and hence it carries a higher risk for psychiatric side effects [7]. Ketamine is commercially available as a racemic mixture in the US, while the S(+) ketamine formulation is available in Europe [9]. Although primarily classified as an NMDA receptor antagonist, its ability to bind to multiple receptors has been identified.

Pharmacokinetics

Ketamine can be safely administered through multiple routes with variable bioavailability. In its intravenous form, ketamine has a bioavailability of 100% with an onset of action of 30 seconds, and when given intramuscularly, ketamine has a bioavailability of roughly 90% [10], which is significantly reduced in its oral (17%) or rectal (25%) form. The reason for this difference is related to its extensive first-pass metabolism. Intranasally, ketamine has a bioavailability of around 50% and is the preferred form among recreational users. As it is a lipid-soluble drug with a low binding to plasma proteins, ketamine has an extensive volume of distribution and is absorbed by the brain quickly with a distribution half-life of 10-15 minutes and a distribution volume of around 2.3 l/kg [2,11].

Once in the body, ketamine is primarily metabolized by the cytochrome system of the liver into several metabolites, the most prominent one being norketamine. The cytochrome P450 3A4 is the principal cytochrome involved in its metabolism. Conditions affecting hepatic metabolism may reduce ketamine clearance. Norketamine is an active metabolite and has one-third of the anesthetic potency of ketamine, and its activity may lead to decreased dose requirement for ketamine during long-term use [7,11,12]. The norketamine will be further hydroxylated before being excreted in bile and urine. Roughly 90% of the drug will be excreted in urine and the rest in feces, with a half-life of two to three hours [13].

Mechanism of Action

Ketamine has a complex mechanism of action involving multiple neurotransmitters. It is a non-competitive inhibitor of the NMDA receptor. This non-competitive binding (Figure 1) is responsible for its various properties including amnesia, psychosensory property, analgesia, and neuroprotection. Studies have identified additional binding sites to the opioid, monoaminergic, nicotinic, and muscarinic receptors as well [6,14].

The NMDA receptor is the most abundant excitatory receptor in the central nervous system (CNS). Glutamate is its principal ligand, and it requires the activity of glycine as a co-agonist and is partially inhibited by magnesium [14]. Ketamine binds to two different sites of the NMDA receptor. By binding to the phencyclidine site, ketamine prevents the regular influx of ions during glutaminergic synaptic transmission, leading to a decrease in the duration of the channel opening. When it binds within the hydrophobic domain, it decreases the frequency of the channel opening [6,11]. A drug-trapping model was initially postulated for ketamine. As glutamate dissociates and closure of the channel occurs, ketamine remains trapped within the channel and continues the channel blockade.

As an NMDA antagonist, ketamine may have a neuroprotective role against glutamate-induced neurotoxicity, caused by prolonged activation of NMDA receptors [15]. This effect has been associated with a reduction in neuronal damage following status epilepticus and improved neuronal survival in stroke and neurotrauma, in animal models [16]. Other animal models have also shown that neuroprotection could be related to the inhibition of dephosphorylation of neuronal-specific cAMP response element-binding protein (CREB) at the perilesional area [17].

Besides the NMDA receptor, ketamine binds to all the opioid receptors with variable affinity [6], having the highest affinity to the mu receptors [18]. Ketamine has been shown to lead to the regulation of central antinociceptive mechanisms in animal models [19]. Ketamine binds to GABA-A receptors in the spinal cord where it has been shown to potentiate the inhibitory effect of GABA [2]. Ketamine also binds with muscarinic receptors and causes dose-dependent blockade of its properties. This peripheral anticholinergic effect can explain its bronchodilation and can also partially explain its sympathomimetic properties [20]. The central sympathomimetic properties of ketamine are related to its NMDA blockade causing the blockade of monoaminergic transporters [21]. The resultant failed reuptake of monoamines causes higher circulating levels of norepinephrine, dopamine, and serotonin levels. Ketamine has recently been shown to block hyperpolarization-activated cyclic nucleotide (HCN) channels, altering cortical neuron electroresponsive properties, which contribute to a ketamine-induced hypnotic-dissociative state [2].

Due to this complex pharmacology, ketamine has demonstrated variable side effects. Most side effects are dose-dependent and are self-limiting upon discontinuation and include hypersalivation, hyperreflexia, transient clonus, vestibular symptoms including dizziness, nausea, vomiting, tachycardia, hypertension, ulcerative cystitis, and detrusor muscle overactivity. During the emergence from anesthesia, the side effects include reactions like hallucination, confusion, combativeness, and impulsiveness and are usually treated with benzodiazepine [2].

Cerebral effects of ketamine

Ketamine has been shown to have extensive effects on several neurophysiologic parameters. Domino et al. described the effect of ketamine on EEG changes during its first experiment in humans. Doses between 0.5 and 2.0 mg/kg have been shown to cause changes in the brain's electrical activity. The most consistent finding was the ability to abolish alpha waves and promote theta activity. This effect was variable depending on the type of enantiomer. The S(+) configuration and racemic mixtures have been shown to decrease EEG amplitude and polymorphic delta activity in a dose-dependent manner. On the contrary, the R(-) configuration does not suppress the EEG pattern [22].

Rat animal models showed an increase in local cerebral glucose utilization and cerebral blood flow, particularly in limbic structures, after treatment with ketamine. These effects are related to increased metabolic activity and activation of neuronal pathways [23].

The initial studies of ketamine had shown an increase in intracranial pressure (ICP), albeit transient, following its use. Initial observations from Shapiro et al. showed that there is a transitory increase in ICP not linked to changes in the partial pressure of oxygen (PaO_2_) or partial pressure of carbon dioxide (PaCO_2_) [24]. During these observations, the data suggested that this increase is likely linked to changes in cerebral blood flow. These observations led to widespread concerns about ICP elevation secondary to ketamine use that discouraged its use in the neurological patient population for a long time. During this time, several studies published findings contradicting this belief, but overall consensus remained against using ketamine for neurological conditions. Pfenninger et al. tested it in animal models, showing no evidence of increased ICP with doses between 0.5 and 2 mg/kg [25]. More recently, a systematic review [26] based on 16 articles that included 127 adult patients did not show any increase in ICP value with ketamine in non-traumatic neurological pathology. Another systemic review [26] by the same group included 101 adult patients from seven articles and found similar results in TBI patients. Based on these findings, there now exists Oxford level 2b and Grade C evidence to support the idea that ketamine does not increase ICP in any neurological condition.

Studies have shown a mass of neuronal and glial depolarization that propagates through the brain following an injury to the human cortex. Leão first described this phenomenon in 1944 in animal models, and it was validated by Strong et al. [27] in 2002. The phenomenon is called cortical spreading depolarization (CSD), which involves waves that occur due to the loss of ions gradients across the cell membrane. This has been recognized based on sustained NMDA receptor activation from acute injury. Hertle et al. [28] showed that ketamine was associated with a decrease in CSD incidence in TBI, SAH, and malignant hemispheric stroke.

Role in neurology

Due to its multiple mechanisms of action and overall favorable effect on neurophysiologic parameters (bolstered by disproven theories about its detrimental effect on ICP), ketamine has reemerged as a medication of interest in neurology. There has been growing evidence to endorse its benefits in a variety of neurological conditions including epilepsy, stroke, migraine, and TBI.

Migraine

Ketamine has been shown to have a role in the treatment of many types of pain. A small open-label RCT of 17 patients by Nicolodi and Sicuteri [29] demonstrated marked pain relief in migraine attacks upon ketamine administration as compared to placebo. However, a subsequent double-blinded RCT by Etchison et al. [30] failed to demonstrate any difference in pain relief between placebo and ketamine groups. Thus, the evidence for the use of ketamine as an abortive treatment for migraine is conflicting and weak at best. On the contrary, there has been growing interest in ketamine as a useful treatment option for medically refractory migraine as well as migraine with aura. A retrospective study of 77 patients by Pomeroy et al. [31] concluded that 71% of patients with refractory migraine experienced short-term relief after multi-day subanesthetic ketamine infusion. Ketamine was started at 0.1 mg/kg/h and was titrated to a maximum dose of 1 mg/kg/h to achieve pain relief. Ketamine reduces the response to repetitive painful stimuli, the “wind-up” effect, in animal models, which is thought to be an important cause of central sensitization and chronic pain in disorders such as migraine. Additionally, prolonged ketamine infusion is associated with the accumulation of norketamine, a key ketamine metabolite, which seems to have a stronger analgesic action. Some preclinical studies [32] have shown that ketamine may inhibit the calcium-mediated release of calcitonin gene-related peptide (CGRP) in trigeminocervical complex, which has been shown to play an important role in the central nociceptive mechanism. However, human data regarding this mechanism of ketamine is lacking. Several clinical and neuroimaging findings support the concept of CSD as the pathophysiological correlate of the neurological symptoms in migraine aura. Ketamine, by its anti-glutamate action, seems to block CSD in animal models [33]. A double-blinded RCT in 2013 compared intranasal ketamine to midazolam in the treatment of migraine with aura, and the ketamine group showed a significant reduction in the severity of aura [34]. Ketamine seems to hold a promise for the management of refractory migraine and migraine with aura, which needs to be thoroughly replicated in larger studies.

Status Epilepticus

Status epilepticus is a neurological emergency with significant morbidity and mortality. Its annual incidence ranges from 5 to 40 per 100,000 people. Status epilepticus has been associated with a failure of the seizure termination mechanism. RSE has been defined as a persistent status epilepticus despite the administration of one first-line and one second-line medication [35]. During RSE, there is a downregulation of the GABA receptors, which can lead to the failure of commonly used anti-epileptic agents. This process is facilitated by the sustained activation of the NMDA receptors and subsequent glutamate excitotoxicity [36]. Ketamine, due to its anti-glutaminergic action, may have a role in the treatment of RSE cases.

Gaspard et al. [37] have shown that ketamine is a safe and possibly efficacious agent in the treatment of RSE based on their multicenter retrospective study of 58 patients. They used ketamine doses as high as 10 mg/kg/h without any adverse events in this study. A large meta-analysis by Rosati et al. [38] included 238 adult patients who received ketamine for seizure control and found ketamine to have an efficacy rate as high as 70% in achieving RSE control. Ketamine infusion was used in the dose range of 0.07-15 mg/kg/h among these patients. Another single-center observational study involving 68 super-refractory status epilepticus (SRSE) patients showed that ketamine infusion achieved a reduction in seizure burden by at least 50% within 24 hours in 81% of patients, with complete cessation in 63% of patients [39]. In this study, ketamine was used as the second anesthetic agent after the failure of seizure control with the first agent (commonly midazolam). The average ketamine infusion rate was 2.2 ± 1.8 mg/kg/h and the duration ranged from one to four days. The Neurocritical Care Society's status epilepticus guidelines from 2012 [40] acknowledged ketamine as an emerging therapeutic option based on the then available data. Since then, multiple observational studies and systemic reviews, like those mentioned above, have reinforced the evidence for its use. The optimal timing and dosing of ketamine in status epilepticus need to be determined in future multicenter randomized studies.

Traumatic Brain Injury

Due to initial concerns about its role in the increase in ICP, ketamine was avoided as an anesthetic agent in TBI for a long time. However, multiple studies have disproven this idea, and there has been a resurgence in the use of ketamine in TBI. A recent systematic review [41] including 11 studies evaluated the use of ketamine in a specific TBI population and found no persistent ICP elevation with ketamine use. Only one study in this analysis found an intermittent elevation in ICP, but cerebral perfusion pressure (CPP) values remained constant. More importantly, there was no evidence of any harm caused by ketamine in any of these studies. Interestingly, three of the 11 studies showed a decrease in CSD with the use of ketamine in a dose-dependent manner.

On the other hand, the choice of this agent in TBI patients requiring analgesia and/or sedation is not well described as larger randomized studies have excluded patients with neurological injuries. The Brain Trauma Foundation guidelines do not recommend any specific agent due to this reason. Ketamine, in addition to its analgesic and sedative effects, has anti-seizure properties as well as a positive effect on hemodynamic values and airway resistance. These properties can be useful in the TBI population that has a high incidence of post-traumatic seizure and often suffer from concurrent hemorrhagic shock and lung trauma. Such versatility and safety of ketamine have raised hopes about ketamine being the answer to the long quest for an effective agent for sedation and analgesia in the TBI population [42].

Stroke

SAH results from the rupture of an arterial aneurysm and is associated with high early mortality and morbidity rates. Delayed cerebral ischemia (DCI) is the main complication of aneurysmal SAH. It can be seen in up to 40% of SAH cases, while radiologically confirmed DCI is noted in 10-13% of SAH cases. The pathophysiology of DCI is multifactorial, related to cerebral vasospasm, neuroinflammation, microthrombosis, and CSD. Ketamine ameliorates CSD, and it increases cerebral blood flow and reduces glutamate-induced neuroinflammation by its primary NMDA receptor-blockade action. Von der Brelie et al. [43] performed a retrospective analysis of 65 SAH patients, out of which 41 received ketamine for sedation; they found a decreased incidence of DCI in the ketamine group (7.3% vs. 25%, p<0.04). The patients receiving ketamine had a higher World Federation of Neurosurgeons Scale (WFNS) grade SAH than the control group and therefore received sedation for a longer duration (mean of 21 days). The benefits against DCI still persisted in the ketamine group. The ketamine dose was titrated to a Richmond Agitation-Sedation Scale (RASS) score of -4 with the maximum dose limited to 500 mg/h. The vasopressor dose was significantly decreased in the ketamine group and there was a trend toward reduction in ICP as well. This protective effect against DCI certainly needs to be investigated further. The upcoming KIND (Ketamine in Neurological Deficit; NCT02636218) trial will use subanesthetic ketamine infusion in SAH patients and will assess its efficacy in DCI prevention.

Similar to SAH, the role of ketamine as a neuroprotective agent is being explored in ischemic stroke as well. Early preclinical data in the mice model showed reduced infarct size post-thrombolytic therapy in ketamine-treated mice [44]. A double-blinded RCT - KETA (Ketamine for Thrombolysis in Acute Ischemic Stroke; NCT02258204) - is designed to assess this effect in humans and is currently recruiting subjects.

Anti-NMDA Receptor Encephalitis

Anti-NMDA receptor encephalitis is an auto-immune disorder associated with the production of antibodies against NR1 and NR2 subunits of the NMDA receptor. Its typical symptoms include psychosis, seizures, memory and cognitive deficits, movement disorders, dysautonomia, and/or decreased level of consciousness. Based on anecdotal case reports and the authors' personal experience, the use of ketamine leads to favorable results in the treatment of movement disorders [45] and status epilepticus [46] in anti-NMDA receptor encephalitis. The use of ketamine, an NMDA antagonist, seems counterintuitive at first thought against a disorder that is known to cause a reduction in NMDA receptors. The observed benefit could be related to the complex pharmacologic action of ketamine on the NMDA receptor itself, other receptor systems (opioid, GABA-A, muscarinic), or reuptake systems (serotonin, norepinephrine, dopamine, and GABA). The use of ketamine for symptomatic treatment in anti-NMDA receptor encephalitis needs further exploration.

Table 1 provides a summary of various studies on the use of ketamine in different neurological disorders.

**Table 1:** Studies on the use of ketamine in different neurological disorders CPP: cerebral perfusion pressure; CSD: cortical spreading depolarization; DCI: delayed cerebral ischemia; ICP: intracranial pressure; MAP: mean arterial pressure; NMDA: N-methyl D-aspartate; RCT: randomized controlled trial; RSE: refractory status epilepticus; SAH: subarachnoid hemorrhage; SRSE: super-refractory status epilepticus; TBI: traumatic brain injury

Study	Design	Number of patients	Target	Ketamine dose	Results
Migraine					
Nicolodi and Sicuteri, 1995 [29]	RCT	17	Acute	0.08 mg/kg	Marked relief
Etchison et al., 2018 [30]	RCT	34	Acute	0.2 mg/kg	No difference
Pomeroy et al., 2016 [31]	Retrospective study	88	Refractory chronic migraine	0.1 mg/kg/h, max. 1 mg/kg/h	Short-term relief in 71% of subjects
Afridi et al., 2013 [34]	RCT	18	Migraine w/aura	25 mg intranasal	Reduced severity of aura compared to midazolam
Status epilepticus					
Gaspard et al., 2013 [37]	Retrospective study	58	RSE	Max. 10 mg/kg/h	57% efficacy, safe agent
Rosati et al., 2018 [38]	Systemic review	238	RSE	0.07–15 mg/kg/h	70% efficacy
Alkhachroum et al., 2020 [39]	Retrospective study	68	SRSE	2.2 ± 1.8 mg/kg/h	Decreased seizure burden in 81%, complete cessation in 63%
Traumatic brain injury					
Gregers et al., 2020 [41]	Systemic review	334	TBI	0.3–6 mg/kg/h	No adverse effect of ICP, no effect on CPP/MAP, reduction in CSD
Subarachnoid hemorrhage					
Von der Brelie et al., 2017 [43]	Retrospective study	65	SAH	Max. 500 mg/h	Decreased incidence of DCI (7.3% vs. 25%)
Anti-NMDA receptor encephalitis					
MacMohan et al., 2013 [45]	Case report	1	Dyskinesia	20 mg/h	Improved dyskinesia in encephalitis
Santoro et al., 2019 [46]	Case report	3	Status epilepticus	40–50 mg load, 3 mg/kg/h infusion	Complete cessation of seizures

## Conclusions

Ketamine is a fascinating anesthetic agent that has gained immense popularity among many medical specialties. It works on multiple CNS receptor systems and exerts complex neurological interactions. Once widely misspread, the belief that ketamine causes ICP elevation has been disproven, leading to its resurgence in the field of medicine. It has the potential for widespread application in the field of neurology, including in the treatment of status epilepticus, migraine, SAH, TBI, and autoimmune encephalitis. The ongoing trials and emerging data will help us cement our knowledge about its use in the coming years.
